# Tissue inhibitor of metalloproteinase 4 in aqueous humor of patients with primary open angle glaucoma, pseudoexfoliation syndrome and pseudoexfoliative glaucoma and its role in proteolysis imbalance

**DOI:** 10.1186/1471-2415-13-69

**Published:** 2013-11-08

**Authors:** Nikitas Fountoulakis, Georgios Labiris, Antonios Aristeidou, Andreas Katsanos, Ioannis Tentes, Alexandros Kortsaris, Vassilios P Kozobolis

**Affiliations:** 1 Department of Ophthalmology, Democritus University of Thrace, Alexandroupolis, Greece; 2 Eye Institute of Thrace, Alexandroupolis, Greece; 3 Department of Ophthalmology, University of Ioannina, Ioannina, Greece; 4 Department of Biochemistry, Democritus University of Thrace, Alexandroupolis, Greece

**Keywords:** Glaucoma, Pseudoexfoliation, Metalloproteinases, TIMP4, TIMP/MMP ratios

## Abstract

**Background:**

To quantify the levels of tissue inhibitor of metalloproteinase 4 (TIMP4) and its ratios with free metalloproteinases (MMP) in the aqueous humor of patients with primary open angle glaucoma (POAG), pseudoexfoliation syndrome (PXS) and pseudoexfoliative glaucoma (PXG) and to evaluate a possible imbalance between MMPs and TIMPs in these samples.

**Methods:**

Free MMP2, MMP3, MMP9, TIMP1, TIMP2, TIMP4 concentrations and active levels of MMP2 and MMP3 were determined with immunoassay ELISA and activity assay kits in 168 aqueous samples.

**Results:**

TIMP4 was elevated in glaucoma patients(POAG: 0.95 ± 0.49 PXG: 1.28 ± 1.38 pg/ml. p < 0.001). POAG, PXS and PXG samples demonstrated higher MMP2, TIMP1 and TIMP2 concentrations (p < 0.001). Samples from the PXS and PXG groups had a lower total/active MMP2 ratio (p < 0.004 and p < 0.008 respectively). Stoichiometric analysis showed an overbalance of TIMPsover MMPs in both POAG & PXG groups,especially of TIMP4.

**Conclusion:**

TIMP4 elevation is a novel finding in glaucomatous eyes. A disregulation of extracellular matrix homeostasis is suggested in POAG, PXS and PXG.

## Background

The most important risk factor for the development and progression of glaucoma is the elevation of the intraocular pressure (IOP) [1]. IOP regulation is directly associated with aqueous humor outflow. Although the exact mechanism has not been thoroughly elucidated, studies have shown that the main resistance to the aqueous humour outflow is located at the trabecular meshwork (TM), especially the juxtacanalicular part directly underneath the inner wall of Schlemm’s canal [2,3]. The special characteristics of the extracellular matrix and the cell interactions in this region can potentially determine the outflow facility [4].

In glaucoma, the normal architecture and function of the juxtacanalicular TM is altered by the pathological accumulation of connective tissue and the partial loss of endothelial cells [5,6]. Other local factors such as the expression of vasoconstrictive molecules and oxidative stress may also be involved [7,8].

Pseudoexfoliation syndrome (PXS) is an age-related systemic disorder characterized by the abnormal accumulation of extracellular matrix (ECM) material in extraocular and ocular tissues. The pseudoexfoliative material might congest the outflow pathways of the TM contributing to the elevation of the IOP and may be involved in the development of pseudoexfoliative glaucoma (PXG) [9,10].

ECM remodeling is primarily controlled by endogenous enzymes, known as matrix metalloproteinases (MMPs). MMPs are a family of endopeptidases that contain an active Zn2+ site which is responsible for their enzymatic activity [11]. They are synthesized as pro-enzymes and secreted as inactive molecules which are activated in vivo by disruption of the cystein-Zn link [11,12]. The regulation of the proteolytic activity of MMP is mainly controlled by the presence of tissue inhibitors of MMPs (TIMPs) which bind MMPs in a 1:1 stoichiometry [13]. Four TIMPs have been identified in vertebrates which can inhibit the action of MMPs. All TIMPs except TIMP3 are in soluble form [13,14]. In biological fluids, MMPs are found either as free proforms, activated enzymes or as complexes with TIMPs.

It is well documented that MMPs and TIMPs play an important role in many ocular pathological processes, including glaucoma [15]. In vitro and in vivo studies have shown that MMPs in TM directly control outflow resistance [16]. Their presence in aqueous humor has also been established [17,18]. Moreover, the recently identified TIMP4, which is associated with MMP inhibition along with other biological processes such as apoptosis [19], has never been traced or quantified in the aqueous humor.

Within this context, the objectives of this study were: a) to trace and estimate the levels of TIMP4 in the aqueous humor, b) to investigate the ratios of TIMP4 to other free MMPs and TIMPs in the aqueous humor, and c) to investigate the potential imbalance of free forms of MMPs and TIMPs in eyes with POAG, PXS and PXG.

## Methods

### Setting

This was a clinic-based, cross-sectional study. The protocol adhered to the tenets of the Helsinki Declaration and written informed consent was given by all participants. The institutional review board of the Democritus University of Thrace approved the protocol and the study was conducted at the Department of Ophthalmology at the University Hospital of Alexandroupolis, Greece.

### Participants

Participants were recruited from the outpatient service of the Department of Ophthalmology at the University Hospital of Alexandroupolis, Greece, in a consecutive-if-eligible basis. All participants were divided into 3 study groups: POAG, PXS, and PXG. The diagnosis of glaucoma and pseudoexfoliation syndrome was made by a glaucoma specialist (GL or VPK) after an evaluation that included slit-lamp examination with mydriasis, gonioscopy, tonometry, central corneal thickness measurement, visual field testing and optic disc assessment with a 90 diopters non-contact lens. Visual field defects were assessed with Humphrey perimetry, using the SITA Standard 30–2 program. The diagnosis of glaucoma and pseudoexfoliation syndrome were made according to the European Glaucoma Society guidelines [20].

Exclusion criteria for all participants were: a) history of optic neuropathy other than glaucoma, b) retinal pathology, c) corneal opacity, d) penetrating eye surgery or other ocular operations any time in the past, e) previous ocular inflammation and f) systemic disorders such as diabetes mellitus and autoimmune or inflammatory diseases that could have affected the optic nerve. All study groups were compared with an age-matched sample of control participants (CG) recruited from the list of patients scheduled for routine cataract surgery after an examination by a glaucoma expert (GL). Controls had to have no other ocular pathology except age-related cataract. They were also excluded if they had any of the systemic disorders listed above. Exclusion criteria for the PXS group were: IOP > 21 mmHg at any time point according to the participant’s medical records, optic disc suspicious for glaucoma, visual field defects and the use of antiglaucoma medications.

### Sample collection

All aqueous humor samples were collected by the same surgeon (VPK) at the initial steps of scheduled cataract or combined cataract-trabeculectomy surgery. Preoperative chemoprophylaxis included only topical use of 5% povidone iodine solution for 30 seconds immediately before the operation. Anterior chamber paracentesis was made through clear cornea with a 27G tuberculin syringe avoiding contact with other ocular tissues. The samples (mean quantity 150 μl) were stored in -73° Celsius. After collecting the aqueous humor sample, the routine steps of the planned operation were performed.

### Quantitative analysis

In order to assess the free total (proform and active) concentrations of MMP2, MMP3 and MMP9 in aqueous humor, 96 well enzyme immunoassay ELISA kits were used (Quantikine; R&D Systems, Minneapolis, MN). Similar ELISA kits (Quantikine; R&D Systems, Minneapolis, MN) were used to determine the concentration of total TIMP1, TIMP2 and TIMP4. All ELISA measurements included free forms of molecules. The quantification of active MMP2 and MMP3 was performed with activity assay kits (Biotrak,Amersham Biosciences, GE Healthcare). The latter quantified endogenously activated enzyme levels and total levels (proform and active) without taking into account complexed forms. This strategy may have helped increase the sensitivity and validity of the study. Sample preparation, dilution and enzyme detection were performed according to the specific assay instructions. More specifically, a 10-fold dilution was used for MMP2, MMP3, TIMP2 and TIMP4, while a 100-fold dilution was used for TIMP1. MMP9 quantification required both 10- and 100-fold dilutions in order to confirm the results. The aqueous quantity added to each well was appointed by each ELISA kit (Table 1).

**Table 1:** Detection sensitivity for Quantikine ELISA and Activity Assay Kits (Manufacturer’s data)

**MMP2**	0.016 – 0.289 ng/mL (mean 0.047 ng/mL)
**MMP3**	0.002 – 0.045 ng/mL ( mean 0.009 ng/mL)
**MMP9**	0.156 ng/mL
**TIMP1**	0.08 ng/mL
**TIMP2**	0.004 – 0.064 ng/mL (mean 0.011 ng/mL)
**TIMP4**	2.14 – 10.0 pg/mL (mean 4.91 pg/mL)
**Active MMP2**	0.19 ng/mL (increased sensitivity protocol)
**Active MMP3**	0.1 ng/mL (increased sensitivity protocol)

MMP: Matrix metalloproteinase, TIMP: Tissue inhibitor metalloproteinase.

Graphic curves were designed using the Origin 6 software (OriginLab Corporation) according to sample optical densities and concentrations. After averaging the duplicate readings for each standard, control and sample, the average zero standard optical density was subtracted. By plotting the optical density for the standards versus the concentration of the standards, the best curve was drawn. The corresponding concentration for each enzyme was calculated, after multiplication by the dilution factor. The detection sensitivity of each kit is presented in Table 1.

### Statistical analysis

The SPSS statistical software (SPSS Inc, Chicago, IL, v.13) was used for the analysisof results. Sample normality was assessed with the Kolmogorov Smirnoff test. Multiple independent samples tests were performed with the Kruskal-Wallis test and confirmed by two independent Mann–Whitney rank sum tests. All tests were two-tailed and statistical significance was considered for p < 0.01 after Bonferroni correction.

## Results

One hundred and sixty eight eyes from 168 white participants (56% men, mean ± SD age: 75 ± 7.6 years, range: 36 to 88 years) were included. There was no statistical difference in the mean age of the groups. Demographic and clinical parameters are shown in Table 2. Quantitative differences in MMPs and TIMPs are presented in Table 3.

**Table 2:** Clinical parameters for the study groups

	**Control**	**POAG**	**PXS**	**PXG**
	**(n = 44)**	**(n = 36)**	**(n = 44)**	**(n = 44)**
**AGE(years)**	75.27 ± 5.66 (63–86)	75.74 ± 7.4 (59–86) (p = 0.96)	75.76 ±5.15 (61–84) (p = 0.96)	75.96 ± 6.37 (64–88) (p = 0.96)
**IOP (mm Hg)**	14 ± 2.35 (9–17)	19 ±6.6 (10–40)	14.7 ± 3 (9–20)	21.1 ±9,3 (8–38)
**Optic nerve c/d ratio**	0.25 ± 0.053 (0.1–0.4)	0.62 ± 0.17 (0.4-0.9)	0.28 ±0.071 (0.2–0.4)	0.68 ± 0.16 (0.4–0.9)
**Number of topical medications**	nil	1 (49%)	nil	1 (9%)
		2 (23%)		2 (13%)
		3 (11%)		3 (47%)
		4 (6%)		4 (22%)

POAG: Primary open angle glaucoma, PXS: Pseudoexfoliation, PXG: Pseudoexfoliation glaucoma, c/d: cup-to-disc ratio. Results are presented as mean ± SD (range).

p values refer to the comparison between each study group versus controls with the Kolmogorov-Smirnoff test.

**Table 3:** Levels of total free (proform/active), active matrix metalloproteinases and tissue inhibitor metalloproteinases in aqueous humor

	**Control**	**POAG**	**PXS**	**PXG**
**MMP2**	7.13 ± 1.5 (5.3-10.2)	23.7 ±10.5 (7.16-44) (p < 0.001)*	15.8 ± 9.8 (6.1-29.3) (p < 0.001)*	23.4 ±17.3 (7.1-56.7) (p < 0.001)*
**MMP3**	3.11 ±0.8 (1.6-4.5)	7.5 ±15.8 (1.3-61) (p = 0.07)	6.8 ± 11.7 (1.3-44.,2) (p = 0.11)	9.8 ± 16.8 (1.5-72) (p < 0.001)*
**MMP9**	0.45 ± 0.25 (0.2-0.97)	0.7 ± 0.6 (0.2-2.1) (p = 0.08)	0.6 ± 0.33 (0.2-1.3) (p = 0.2)	0.6 ± 0.35 (0.17-1.5) (p = 0.2)
**TIMP1**	16.3 ± 6.1 (9.3-33.5)	33.6 ± 19.3 (11–75) (p < 0.001)*	31.2 ± 14.5 (12–58.5) (p < 0.001)*	37 ± 15 (15–60.3) (p < 0.001)*
**TIMP2**	3.7 ± 1.1 (2–5.7)	6.5 ± 3.1 ( 2.1-13.2) (p < 0.001)*	5.3 ± 2.1 (2.4-8.9) (p < 0.001)*	6.3 ± 3.1 (2.3-12.2) (p < 0.001)*
**TIMP4**	0.48 ± 0.25 (0.22-0.98)	0.95 ± 0.49 (0.24-1.76) (p < 0.001)*	0.48 ± 0.2 (0.13-0.76) (p = 0.93)	1.28 ± 1.38 (0.4-5.9) (p < 0.001)*
**Active MMP2**	0.21 ± 0.01 (0.2-0.24)	0.27 ± 0.06 (0.2-0.42) (p < 0.001)*	0.24 ± 0.03 (0.19-0.3) (p < 0.003)*	0.25 ± 0.04 (0.19-0.33) (p < 0.001)*
**Active MMP3**	0.29 ± 0.01 (0.26-0.3)	0.32 ± 0.02 (0.28-0.37) (p < 0.001)*	0.32 ± 0.01 (0.3-0.35) (p < 0.001)*	0.32 ± 0.2 (0.29-0.35) (p < 0.001)*
**Act/tot. MMP2**	0.008 ± 0,00086	0.006 ±0,00279 (p = 0.03)	0.005 ±0,00234 (p = 0.004)*	0.005 ± 0,00299 (p = 0.008)*
**Act/tot. MMP3**	0.008 ± 0,00183	0.008 ± 0,00061 (p = 0.93)	0.007 ± 0,0023 (p = 0.1)	0.008 ± 0,00072 (p = 0.93)

MMP: Matrix metalloproteinase, TIMP: Tissue inhibitor metalloproteinase, POAG: Primary open angle glaucoma, PXS: Pseudoexfoliation, PXG: Pseudoexfoliation glaucoma. Concentrations (ng/mL) are presented as mean ± SD(range).

p values refer to the Kruskal-Wallis multiple sample test for each study group versus controls. Statistical significance: p < 0.01 after Bonferroni correction.

*: statistically significant.

### TIMP4 levels

TIMP4 levels were higher in both glaucoma groups (POAG and PXG) when compared to controls (p = 0.001). In contrast, in the PXS group, TIMP4 concentrations presented no statistical difference from controls (p = 0.936) (Table 3).

### Free MMP levels – other TIMP levels

Further analysis was extended to other free MMPs and TIMPs. In POAG, PXS and PXG samples, the concentrations of total free MMP2, TIMP1 and TIMP2were significantly higher compared to controls. In PXG eyes, free MMP3 was also elevated (p < 0.001) compared to controls (Table 3).

### Activity assays

POAG, PXS and PXG patients had higher levels of active MMP2 (p < 0.001, p = 0.003 and p < 0.001, respectively) and active MMP3 (p < 0.001) than controls. PXS and PXG samples though, had lower ratios of active/total MMP2 (p = 0.004 and p = 0.008) (Table 3).

### MMP/TIMP ratios in different study groups

In order to evaluate a possible imbalance in ECM modulation, a thorough evaluation of MMP/TIMP ratios was performed.

POAG samples showed a decreased ratio of MMP-9/TIMP-4 compared to controls. In this group, we found lower ratios of MMP-3 and MMP-9 to the other TIMPs compared to controls (Table 4).

**Table 4:** MMP/TIMP ratios for the study and control groups

	**CONTROL**	**POAG**	**PXS**	**PXG**
**MMP2/TIMP1**	0,464 ± 0,1	0,805 ± 0,31 (p > 0.1)	0,73 ± 0,753 (p > 0.1)	0,618 ± 0,360 (p > 0.1)
**MMP2/TIMP2**	2,07 ± 0,65	4,04 ± 2(p = 0.069)	2,8 ± 1,09 (p = 0.06)	4,34 ± 3,59 (p = 0.06)
**MMP2/TIMP4**	18,42 ±8,4	33,54 ± 25,61 (p = 0.031)	34,47 ± 19,07 (p < 0.001)*	28,36 ± 26,88 (p = 0.73)
**MMP3/TIMP1**	0.21 ± 0,085	0.17 ± 0.2 (p = 0.001)*	0.226 ± 0.4 (p < 0.001)*	0.26 ± 1.15 (p = 0.02)
**MMP3/TIMP2**	0.95 ± 0.48	0.88 ± 1.16 (p = 0.007)*	1.68 ± 3.17 (p = 0.01)*	2.07 ± 3.9 (p = 0.39)
**MMP3/TIMP4**	8,47 ± 4,86	11,93 ± 24,07 (p > 0.1)	14,16 ± 31,4 (p > 0.1)	16,85 ± 40,98 (p > 0.1)
**MMP9/TIMP1**	0.028 ± 0.014	0.023 ± 0.017 (p < 0.001)*	0.02 ± 0.019 (p < 0.001)*	0.02 ± 0.018 (p < 0.001)*
**MMP9/TIMP2**	0.13 ± 0.07	0.104 ± 0.059 (p < 0.001)*	0.12 ± 0.06 (p < 0.001)*	0.104 ± 0.063 (p < 0.001)*
**MMP9/TIMP4**	1.18 ± 0.99	1,12 ± 1.08 (p = 0.01)*	1.42 ± 0.7 (p = 0.4)	0.8 ± 0.59 (p < 0.001)*

MMP: Matrix metalloproteinase, TIMP: Tissue inhibitor metalloproteinase, POAG: Primary open angle glaucoma, PXS: Pseudoexfoliation, PXG: Pseudoexfoliation glaucoma.

Values are presented as mean ± SD.

p values refer to the Mann Whitney test for each study group versus controls. Statistical significance: p < 0.01 after Bonferroni correction.

*: statistically significant.

PXG samples presented similar ratio results as POAG samples, although the differences in MMP3 to other TIMPs ratios did not reach statistical significance (Table 4).

In PXS eyes, an increased ratio of MMP2/TIMP4 was found. The other MMP/TIMP ratios are showed in Table 4.

### Differences between study groups

Compared to PXS samples, PXG samples demonstrated higher TIMP4 concentrations (p < 0.001), lower TIMP4 ratios [MMP2/TIMP4 (p < 0.001) and MMP9/TIMP4 (p < 0.001)] and higher MMP3 levels (p = 0.008). No differences between POAG and PXG eyes were detected.

## Discussion

MMPs play a critical role in TM tissue remodeling. They do not only function as catalytic proteases, but also as extracellular processing enzymes that influence cell- to-cell and cell-to-matrix signaling [21]. Their action is regulated by gene expression, pericellular accumulation, biochemical activation and interaction-deactivation with TIMPs, their endogenous inhibitors. Although the action of MMPs involves a large variety of connective tissue elements, their secretion depends on the specific substrate needed to be resolved. Several studies showed that in a given environment, the concentration of active enzyme may be directed by the concentration of a preferred ECM element, since specific MMPs degrade some substrates more efficiently than others [22-24]. The cells keep a high reserve of surface-anchored MMPs which are released as free molecules when the pericellular environment biochemically permits allosteric or other types of activation [21]. Thus, free MMPs in a cellular environment reflect ready-for-action molecules.

Glaucoma and MMP regulation have been highly associated since most of the proposed pathogenic mechanisms suggest enzyme dysfunction [15,25]. The presence of MMPs and TIMPs has been established both in the TM and aqueous humor in normal and pathologic conditions. In the aqueous humor, the amount of proteolytic activity and inhibition may reflect the ECM state of remodeling from surrounding anterior segment tissues. MMPs and TIMPs could originate from anterior chamber structures such as the TM and corneal endothelial cells, or from a blood-aqueous barrier breakdown [17]. Although the exact origin is not known, it has been suggested that in glaucoma, the main reason for increased enzymatic levels is local tissue up-regulation [17].

ECM disorders within the TM and surrounding tissues concur in POAG, PXS and PXG [17,18]. In PXG, there is a relationship between PEX material accumulation and glaucoma development and progression [9]. However, there are eyes with excessive accumulation of exfoliation material that do not develop glaucoma; therefore other predisposing factors leading to glaucoma conversion seem to exist [26].

Recent evidence suggests that trabecular cellular function is compromised especially at the juxtacanalicular and Schlemm’s canal region [27]. In trabecular meshwork specimens, besides endothelial dysfunction, there is significant cell apoptosis [8,28]. Furthermore, both PXS and PXG are characterized by impairment of ocular hemodynamics, suggesting ischemic stress at the anterior ocular segment [10,19,29]. Similar pathological phenomena can occur in other systemic diseases, like cardiovascular disorders, where the recently discovered TIMP4 seems to play a significant role. TIMP4 overexpression is highly associated with the regulation of apoptosis, inflammation-induced cell death and inhibition of endothelial cell migration [19,30,31]. Furthermore, its capacity to block the conversion of pro-MMPs to their active forms supports its role in ECM matrix modulation [32].

In this study, using a recently developed ELISA immunostaining kit, we were able, for the first time, to quantify TIMP4 in aqueous humor samples. We found that TIMP4 was only elevated in glaucoma patients. In PXS patients, TIMP4 levels were statistically similar to controls. Furthermore, both POAG and PXG patients showed a decreased ratio of MMP9/TIMP4, a result not seen in PXS samples. Instead, in PXS patients the MMP2/TIMP4 ratio is increased. POAG and PXG patients had similar MMP2/TIMP4 and MMP3/TIMP4 ratios with controls even though there was an increase in the concentration of MMP2 in the POAG group and MMP3 in the PXG group. The increase in TIMP4 levels seems to compensate (or even over-compensate)for the respective increase in MMP concentrations in eyes with POAG and PXG [32]. Thus, besides all other possible TIMP4 properties, the increased levels of this inhibitor may contribute to the altered balance of TIMPs and MMPs in the aqueous humor of glaucoma patients. Our results may suggest an important link between glaucoma and TIMP4 action.

In POAG, several studies have shown that the levels of total MMP2 and MMP3 and the levels of TIMP1 and TIMP2 are generally up-regulated. Furthermore, in PXS and PXG, total MMP-2, total MMP-3, TIMP-1 and TIMP2 levels in aqueous humor were also significantly elevated compared to samples from conntrols [17,18]. Our study investigated the concentrations of free MMP and TIMP in all study groups. In POAG patients it is found that TIMP1, TIMP2 and MMP2 were statistically elevated compared to controls. Furthermore, activity assays showed that the ratio of active/total free MMP2 and MMP3 were not significantly altered in POAG. PXG patients presented higher free MMP3 levels. PXS and PXG patients also had significantly higher concentrations of free MMP2, TIMP1, TIMP2 and active MMP2 and MMP3but a lower ratio of active/total MMP2. This conclusion agrees with previous reports [17,18] and suggests that even though there is a higher production of ECM catabolic enzymes in POAG, their proteolytic action is insufficient for adequate matrix turnover.

The importance of stoichiometric balance between MMP2/TIMP2 was shown by a number of reports [11,17]. In our investigation, the MMP2/TIMP2 ratio in POAG patients was not increased. Therefore, it can be assumed that the elevation of TIMP2 levels is in essence a compensatory reaction to the elevation of MMP2 levels. The comparison of the MMP/TIMP ratios was extended to all studied molecules, because to a certain extent, all TIMP members can inhibit different MMPs. The analysis showed that despite MMP elevation there were no higher levels of MMP/TIMP ratios; instead decreased ratios of MMP3 and MMP9 overTIMP1 and TIMP2 could suggest a major regulatory role of enzyme inhibitors.

Among the objectives of our study was to reveal potential differences within study groups. A recent study with POAG and PXG patients indicated that MMPs/TIMPs ratios from TMs pecimens are decreased in PXG, while in samples of POAG eyes, the MMP-1/TIMP-1 ratio and the total MMP1 + 2 + 3 + 9/total TIMP1 + 2 + 3ratio were increased when compared to PXG eyes [33]. Moreover, MMP immunostaining analysis of aqueous humor samples of the same patients presented no differences between the two glaucoma groups [33]. Our results also showed similar MMP/TIMP aqueous profile between the two groups.

Regarding the comparison between the PXG and PXS groups, the most important differences were found in the TIMP4 concentrations and ratios. No differences were discovered in active MMPs concentrations, or in MMP9/TIMP1-2 ratios. Another notable difference was that MMP3 levels were significantly elevated in PXG, but not in PXS samples. These results suggest that TIMP4 and MMP3 may have a different regulatory role in pseudoexfoliative eyes with and without glaucoma.

Our study has certain limitations. Firstly, there was a wide range of MMP levels within the study groups. This wide variation may reflect differences in phenotypes, disease stage or disease duration. Secondly, the majority of patients were under treatment with topical medication (i.e. prostaglandin analogues, b-blockers etc.) that could potentially affect aqueous composition and MMP concentration [34].

## Conclusions

Our study suggests that there is an imbalance between MMP-related proteolytic activity and its regulation by members of the TIMP family of enzymes in eyes with POAG and PXG. Our novel finding is the elevation of TIMP4 in the aqueous samples of glaucomatous eyes. TIMP4, as a potential inhibitor of MMP activity, could be implicated in ECM homeostasis disorders in glaucoma. Its further role in glaucoma pathology merits further investigation.

## Competing interests

The authors declare that they have no competing interests.

## Authors’ contributions

NF drafted the manuscript. He was also involved in study design, data collection, quantitative analysis of the samples and result interpretation. GL participated in the design of the study and patient examination. He also reviewed the manuscript for important intellectual content. AA participated in data acquisition and drafted part of the manuscript. AK participated in the analysis and interpretation of results and reviewed the draft for important intellectual content. IT carried out the immunoassays and participated in the analysis of data. Al.K coordinated the laboratory procedures and designed the biochemical approach of the study. VPK conceived of the study and was involved in study design, sample collection and patient examination. He also critically reviewed the manuscript for important intellectual content and gave the final approval of the version to be published. All authors read and approved the final manuscript.

## Pre-publication history

The pre-publication history for this paper can be accessed here:

http://www.biomedcentral.com/1471-2415/13/69/prepub
